# A real-world study of hereditary angioedema patients due to C1 inhibitor deficiency treated with danazol in the Brazilian Public Health System

**DOI:** 10.3389/fmed.2024.1343547

**Published:** 2024-09-06

**Authors:** Alessandra Mileni Versuti Ritter, Suelen Silva, Robson de Paula, Juliana Senra, Fabio Carvalho, Tatiane Ribeiro, Solange Oliveira Rodrigues Valle

**Affiliations:** ^1^IQVIA, São Paulo, Brazil; ^2^Takeda Distribuidora Ltd., São Paulo, Brazil; ^3^Hospital Universitário Clementino Fraga Filho da Universidade Federal do Rio de Janeiro, Rio de Janeiro, Brazil

**Keywords:** hereditary angioedema, attacks, SUS, public setting, healthcare resources, androgens

## Abstract

**Introduction:**

Hereditary angioedema (HAE) due to C1 inhibitor (C1-INH) deficiency is an ultra-rare autosomal dominant inherited disease that affects 1 in 67,000 people in the world. The attacks are based on subcutaneous and submucosal edema that can lead to death if not properly managed. Considering the lack of information on the clinical management of Brazilian patients with HAE, this study aimed to identify and characterize patients with HAE-C1-INH that used danazol prophylactic treatment in the Brazilian Public Health System (SUS) and the healthcare resource utilization (HCRU).

**Methods:**

This was an observational retrospective database study with patients treated with danazol from January 2011 until December 2021 within the SUS. The HAE cohort included patients with 12 years or older with at least one record for ICD-10 D84.1, one claim for danazol record, and at least 6 months of available history in the database.

**Results:**

Our study included 799 patients treated in the SUS, with a mean (SD) age at danazol initiation of 40 years (16). The number of patients with HAE showed a similar distribution over this 10-year period analyzed with the highest number of patients in 2015 (*n* = 509) and 2016 (*n* = 480). A total of 253 (32%) patients had a record of at least one attack. Of those, 45 (17.8%) had at least one procedure HAE-related hospital admission, and 128 (50.6%) had at least one HAE-related hospital admission. The mean (SD) hospitalization length of stay was 5 (8) days. Over 14% (*n* = 36) of HAE patients with attack (*n* = 253) had at least one HAE-related ICU admission.

**Conclusion:**

This database study is the strategy used to allow us to find and describe the characteristics of patients with HAE who use danazol for long-term prophylaxis in the SUS and identify HCRU outcomes of interest such as hospitalizations, inpatient, and outpatient settings. The high rate of attacks, hospitalizations, and general resource uses highlights the necessity to increase awareness of new strategies and accurate approaches to treat HAE patients. Therefore, our findings are important indicators that our health system and guidelines need to be revised and improved to properly diagnose, treat, and assist patients with HAE.

## 1 Introduction

Hereditary angioedema (HAE) due to C1 inhibitor (C1-INH) deficiency is an ultra-rare disease that affects 1 in 67,000 people in the world. It is an autosomal dominant inherited disease (1), mostly resulting in decreased production of C1-INH (type I) and/or functional activity of C1-INH (type II), leading to excessive bradykinin production (2). Although HAE is a genetic disease, approximately 25% of the cases are C1-INH *de novo* gene mutations (3, 4).

All types of HAE have similar clinical characteristics and may be triggered by emotional stress, trauma, surgical procedures, infection, and inflammation (5). The manifestations, also called attacks, are based on subcutaneous and submucosal edema due to the bradykinin’s excessive production, usually affecting the face, tongue, body extremities (hands and feet), and genitals (6). The attacks may be triggered by factors such as stress or trauma or no apparent reason and can last from 2 to 5 days without treatment. Laryngeal edema might be present, resulting in asphyxia with a high risk of death if not properly managed (7). In the gastrointestinal tract, it generates symptoms such as transient edema of the intestinal walls, partial or total intestinal obstruction, and/or accumulation of fluid in the abdominal cavity (8).

HAE-C1-INH types I and II can be diagnosed by measuring serum complement levels including C4 and antigenic and functional levels of C1-INH. The clinical diagnosis of HAE-C1-INH is usually based on the appearance of typical clinical manifestations of the disease, such as non-inflammatory subcutaneous angioedema lasting more than 12 h, abdominal pain of undefined organic etiology lasting more than 6 h, swellings not associated with wheals and which do not improve with anti-histaminergic drugs, and recurrent laryngeal edema (3). The main differential diagnoses of HAE are the other types of angioedema, especially those with chronic or recurrent presentation. The most frequent type of recurrent angioedema is histaminergic, which is usually associated with wheals and can be induced or exacerbated using non-steroidal anti-inflammatory drugs (NSAIDs) (3).

The broad and unspecific disease symptoms and unawareness of this rare disease by the treating physician result in long periods of delayed diagnosis and misdiagnosis, leading to mistreatment (9, 10). The long time elapsed between the first symptoms and diagnosis and the misdiagnoses is multifactorial (11). Reasons for diagnostic delay include underrecognized cases from the doctor or even lack of knowledge about the disease, and also the similarity of symptoms with other diseases (12). An international study with HAE-C1-INH patients showed that patients visited an average of 4.4 physicians before the correct diagnosis and took a mean of 8.3 years for diagnosis (13, 14), but a Brazilian study found almost double the time, approximately 14–18 years between the initial manifestation and the diagnosis (11, 15). Another study also reported a delay in the HEA diagnosis, even in those patients with a familial history (10). In this descriptive, cross-sectional study with prospective data collection of 138 Brazilian patients with HAE, the diagnosis delay was 17.7 ± 12.6 years (10).

The management of HAE-C1-INH is based on acute attacks and prophylactic treatment (long- or short-term use) aiming to prevent the attack occurrence. The HAE-C1-INH attack treatment is focused on either preventing or treating them by using prophylactic treatment. Acute attacks may be treated with efficient drugs: the replacement of the lacking protein with plasma-derived human C1-INH (pdC1-INH) or icatibant acetate. Attenuated androgens, antifibrinolytics, and pdC1-INH are the most used drugs for long-term prophylactic treatment, although new drugs have been developed in recent years. Conventional treatment for allergic angioedema with adrenaline, antihistamine, and glucocorticoids has not been efficacious as the mechanism is bradykinin-mediated. Since 2010, the specific treatment is incorporated into the Clinical Protocols and Therapeutic Guidelines (PCDT [*Protocolos Clínicos e Diretrizes Terapêuticas*]) of the disease and includes danazol for long-term prophylaxis and fresh frozen plasma for acute attacks (1, 16, 17).

Given the rarity of the disease, real-world evidence (RWE) on the clinical management of patients with HAE-C1-INH is scarce. RWE is the analysis of real-world data (RWD) that generates evidence related to patient health status and/or the delivery of healthcare. This evidence can assist public managers in making informed decisions about the incorporation, exclusion, or change of new medicines, products, and procedures, as well as in the preparation and review of PCDT (18).

Considering the lack of information on the clinical management of Brazilian patients with HAE, this study aims to identify and characterize patients with HAE-C1-INH that used danazol prophylactic treatment in the Brazilian Public Healthcare System (*Sistema Único de Saúde* [SUS]).

## 2 Methods

### 2.1 Study design and setting

**Figure 1:**
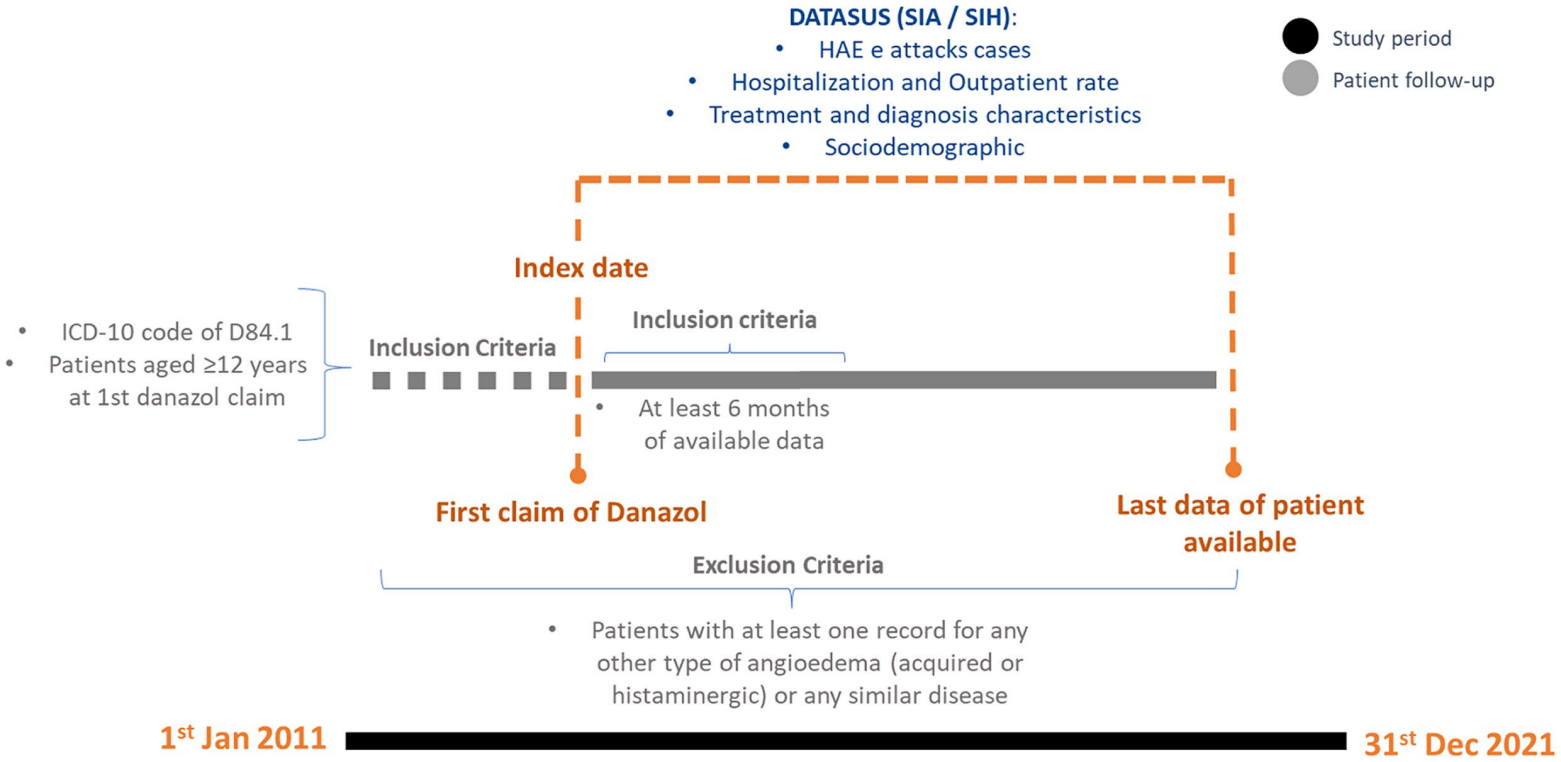
Study design.

This was an observational retrospective database study that aimed to identify, quantify, and characterize patients with HAE-C1-INH treated with danazol from January 2011 until December 2021 within the SUS. Cases were identified based on data gathered from administrative claim databases from the Informatics Department of SUS (DATASUS [*Departamento de Informática do SUS*]). The study design is described in Figure 1.

DATASUS is responsible for processing and disseminating the healthcare data collected in Brazil within the SUS. All the data are collected through a platform that serves for administrative purposes and health indicator tracking. Therefore, DATASUS maintains and generates healthcare information, such as health indicators, healthcare assistance indicators, epidemiologic information, vital statistics, healthcare unit detail registry, demographic, and socioeconomic information. In addition to that, DATASUS is also responsible for financial information in healthcare, such as budget allocation and transfers between the Federal, State, and Municipal levels, reimbursement values, and others.

Specifically, regarding treatment information, DATASUS has a dataset including data on both ambulatory and hospital care at the patient level. DATASUS has information on the procedures performed in SUS, which virtually covers 100% of the Brazilian population. Although there is also a private health system in Brazil, approximately 75–80% of the population rely exclusively on SUS (19).

The administrative claim data are presented as procedure codes from billing records. Each line of the database represents a procedure and includes demographic information, number of procedures such as surgery or any other intervention, costs, and the International Classification of Diseases 10th revision (ICD-10). Data on inpatient and outpatient procedures are publicly available on the DATASUS website, from which we collected all the information used in this study.

In this study, we used the following databases of DATASUS: Inpatient Information System (SIH [*Sistema de Informações Hospitalares*]) and Outpatient Information System (SIA [*Sistema de Informações Ambulatoriais*]). Procedures are organized by the SUS Procedures, Medications and Orthoses, Prostheses and Special Materials Table Management System (SIGTAP [*Tabela de Procedimentos, Medicamentos, Órteses/Proteses e Materiais Especiais do SUS*]) within DATASUS and are comprised by a 10-digit identification number for each procedure available on SUS. Probabilistic record linkage was performed to evaluate subject information, from diagnosis until the last event available in both inpatient and outpatient settings, to describe the patient journey. The probabilistic linkage method used in this analysis was developed by IQVIA, and using different combinations of patient information from both databases, such as date of birth, sex, and ZIP code, identifies patients in both systems. In addition, details regarding this approach are not provided due to confidentiality rights.

### 2.2 Study population

As both SIH and SIA are administrative databases with reimbursement purposes, few clinical data (e.g., signs and symptoms) were available. Because of this, a pre-defined list of causes of admission (ICD-10) and inpatient and outpatient procedures were used to identify patients with HAE, based on the review of the most recent literature available on the disease natural history, diagnosis, treatment, epidemiologic data, and clinical experts’ opinion. The clinical expert who gave input on the criteria to identify the records of HAE patients is an allergologist and clinical immunopathology researcher with more than 20 years of experience in the area and is the reference to the HAE treatment.

The HAE cohort included patients with at least one record for ICD-10 D84.1 (Defects in the complement system C1-INH deficiency)—and at least one claim for danazol after D84.1 record, both from 1 January 2011 to 31 December 2021. Only patients aged 12 years old or more were included in the study since the Brazilian CPTG for HAE-C1-INH treatment excludes children from being treated with danazol. Furthermore, the clinical manifestations, such as recurrent episodes of swelling of affected areas, are more frequent in adolescents and adults compared to children (11). Finally, only patients who used danazol at least once and patients with at least 6 months of available history in the database after the index date were included.

To improve patient selection and decrease misclassification error, we excluded other types of angioedema. We excluded patients with at least one claim of ICD-10 codes that could potentially lead to misclassification (histamine-mediated angioedema and acquired angioedema for non-histamine-mediated angioedema) at any point during the study period. The list of ICD-10 codes that could result in misclassification (used as exclusion criteria) was based on the literature and validated by the clinical expert (Supplementary Table S1).

Since induced angioedema is associated with the use of angiotensin-converting-enzyme inhibitors (ACEi) and other drugs (e.g., NSAID and angiotensin receptor blockers [ARBs], it is not possible to adopt exclusion criteria for this type of angioedema because they are not reported at the SIGTAP).

### 2.3 Study outcomes

The occurrence of procedures/ICD-10 possibly related to HAE attacks was assessed as a primary outcome. To identify HAE attacks within the HAE cohort, we created a preliminary ICD-10 code list (Supplementary Table S2) and procedures (Supplementary Table S3) that could be potentially related to HAE attacks (i.e., ICD-10 codes and/or procedures most commonly presented as symptoms/manifestations of HAE attacks) based on the literature review and clinical expert opinion. Any record of ICD-10 and/or procedures listed in those tables were considered as attack.

Finally, for analysis purposes, the list of ICD-10 codes and procedures pre-defined as attack were segregated as severe and non-severe attack. Thus, an even more restrict list of procedures and ICD-10 codes were used to identify patients with more severe disease and are presented in Supplementary Table S4.

The HAE-related hospitalizations were used as secondary outcome. Hospitalizations were identified by HAE-related claims in the SIH database during the study period. SIH includes the causes of hospitalization according to ICD-10 code. HAE-related hospitalizations were considered as hospital records with the HAE-related ICD-10 codes and procedures (Supplementary Tables S1, S2). Finally, outpatient procedures performed during the study period were assessed considering HAE-related procedures codes recorded in the SIA from the cohort as complement data regarding the resources used.

### 2.4 Statistical analysis

The primary and secondary outcomes were summarized using descriptive statistics, mean, standard deviation (SD), median, interquartile ranges (IQR), and 95% confidence intervals (CI), where applicable. Categorical variables were described using simple and cross-contingency tabulation, with absolute frequencies, percentages, and 95% CIs where applicable.

Demographic characteristics of the study population included gender and age at first danazol claim. The age variable was calculated based on the difference between the date of birth and the first record of the danazol claim (index date). The age was described as a continuous variable, including the mean, standard deviation, median, interquartile ranges, and age groups (absolute number and proportion per category). The demographic variables were described as categorical variables, with absolute frequencies and percentages, as well as the frequency of the selected HAE ICD-10 codes. The time of follow-up was calculated based on the difference between the date of the first claim of danazol (index date) and the last date of patient information available at the database.

HAE attacks were described using absolute (number) and relative (proportion) measurements and were calculated among the total and the attack population by calendar year, and by years after danazol initiation, segregated by severity (severe and non-severe).

Hospitalizations were identified in the SIH database for HAE patients treated with danazol during the study period. HAE-related hospitalizations were considered to estimate the healthcare resource utilization (HCRU) related to attacks and were based on hospital records with the HAE-related ICD-10 codes and procedures (Supplementary Tables S2, S3). The hospitalization length in days was calculated as the difference between the admission and discharge date. This was computed for all patients who had a record of hospitalization. The length of stay was also summarized with mean (SD) and median (IQR). In addition, patients with hospitalization claim(s) were categorized according to the number of hospitalizations in the following range: 1–2 hospitalizations, 3–6 hospitalizations, or 6+ hospitalizations. Those with 6+ hospitalizations in the first year after the index date were further analyzed, and the reasons for hospitalizations (most frequent ICD-10 codes) were listed.

HAE-related healthcare resource utilization of any attack as well as outpatient procedures performed during the study period were assessed as HAE-related healthcare resource utilization. The resource utilization per patient was summarized as the mean (SD) and median (IQR) number of hospital admissions and outpatient visits per each patient; and the resource utilization per patient per year (PPPY) was calculated as the mean and median (95%CI) number of procedures divided by each patient’s follow-up (FUP) time in years, according to the formula:


$$\mathrm{PPPY}=\frac{\mathrm{Nvisitsprocedures}}{FUP\;\mathrm{of}\;\mathrm{each}\;\mathrm{patient}\;\left(\mathrm{in}\;\mathrm{years}\right)}$$

Treatment characteristics were presented as the number and proportion of patients with a record of danazol treatment (prophylactic treatment) or fresh plasma transfusion (on-demand treatment) during the study period and segregated by on-demand and prophylactic treatment.

All analyses, computations and generation of tables, listings, graphics, and data for figures were performed using Python ® version 3.6.9.

## 3 Results

### 3.1 Population characteristics

**Figure 2:**
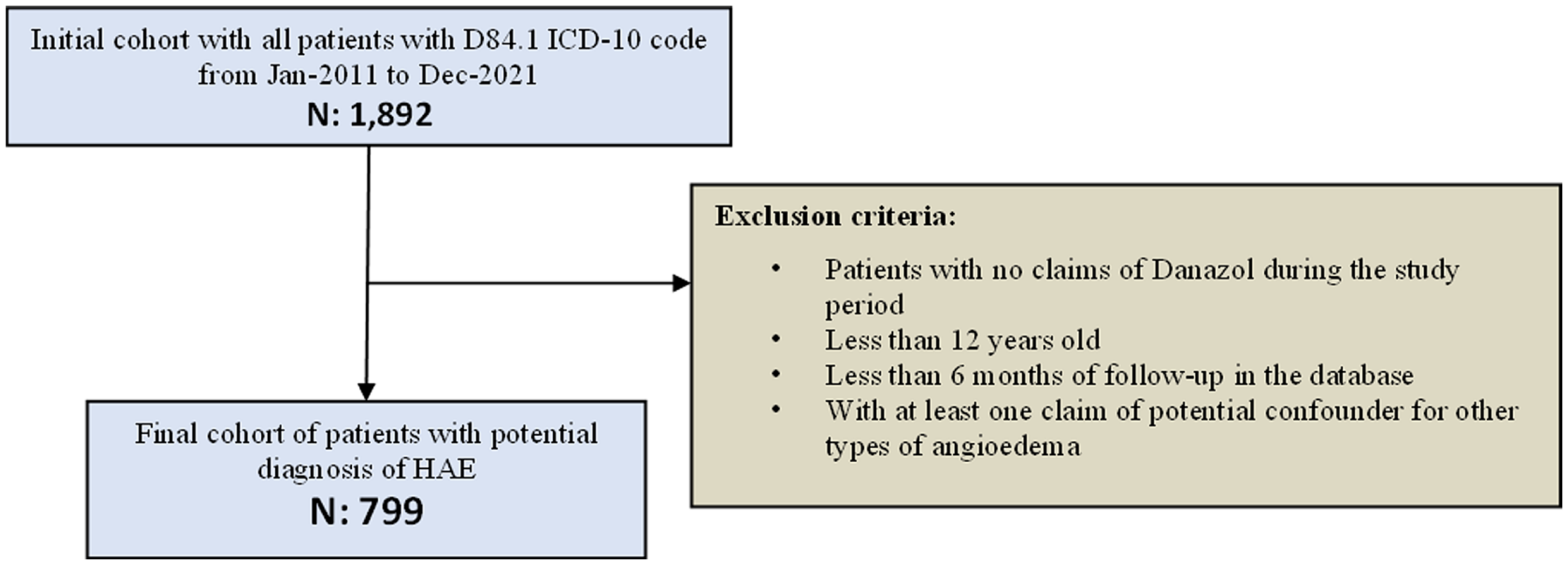
Disposition of subjects.

From January 2011 to December 2021, the SUS database recorded claims for 1,892 patients under the ICD-10 D84.1 code. From these, 1,044 patients had at least one claim of danazol, 1,031 were more than 12-year-old at the index date (first danazol claim), 944 patients had more than 6 months of follow-up at the DATASUS (available data), and 799 patients had no claim of ICD-10 code classified as other types of angioedema. Thus, 799 patients were considered eligible for the study and composed the HAE cohort (Figure 2).

From the 799 patients treated with danazol in the present cohort, most of them were female (65%), the mean (SD) age at danazol initiation was 40 (16) years old, the majority of HAE population was between 21 and 30 years old (21.9%) at index, and the lowest proportion of patients was in extreme ages (younger than 21 years [11.5%] and older than 60 years [12.5%]). Although patients were identified at SUS between 2011 and 2021, each patient contributed with a different follow-up period in the database. The median (IQR) follow-up time of the included patients in the study was 2 (4) years. The prevalence of HAE across the different states of Brazil was heterogenous. Overall, the state with the highest number of HAE patients was São Paulo (43%), followed by Minas Gerais (16%), Paraná (8%), and Rio de Janeiro (8%). There were no patients identified in Amapá, Amazonas, Mato Grosso, Rio Grande do Norte, Rondônia, Roraima, Sergipe, and Tocantins between 2011 and 2021 (Table 1).

**Table 1:** Description of general characteristics of patients with HAE identified at DATASUS between 2011 and 2021 (*n* = 799).

		HAE (*N* = 799)
Follow-up time in years	Mean (SD)	3.06 (2.99)
	Median	2
	Min – Max	0–11
	IQR	4
Age at index date	Mean (SD)	40.06 (16.66)
	Median	38.09
	Min – Max	12.00–98.71
	IQR	24.81
Age group, *N* (%)	12–20 years	92 (11.5%)
	21–30 years	175 (21.9%)
	31–40 years	158 (19.8%)
	41–50 years	145 (18.1%)
	51–60 years	129 (16.1%)
	60+ years	100 (12.5%)
	Unknown	0
Sex (%)	Female	523 (65.5%)
	Male	276 (34.5%)
State of residence	Acre	6 (0.8%)
	Alagoas	1 (0.1%)
	Bahia	38 (4.8%)
	Ceará	1 (0.1%)
	Distrito Federal	16 (2.0%)
	Espírito Santo	51 (6.4%)
	Goiás	6 (0.8%)
	Maranhão	1 (0.1%)
	Mato Grosso do Sul	11 (1.4%)
	Minas Gerais	129 (16.2%)
	Pará	1 (0.1%)
	Paraíba	1 (0.1%)
	Paraná	66 (8.3%)
	Pernambuco	14 (1.8%)
	Piauí	4 (0.5%)
	Rio de Janeiro	60 (7.5%)
	Rio Grande do Sul	52 (6.5%)
	Santa Catarina	43 (5.4%)
	São Paulo	343 (42.9%)
	Amapá, Amazonas, Mato Grosso, Rio Grande do Norte, Rondônia, Roraima, Sergipe, Tocantins, and Unknown	0 (0%)

### 3.2 Danazol treatment characteristics

Table 2 describes all patients identified as HAE cases in DATASUS across the study years (2011–2021). In total, 799 unique patients had at least one claim of danazol. Of those, 281 (35.2%) patients had the first claim of danazol in 2011, and 11 (1.4%) patients had the first claim of danazol in 2021. Between 2012 and 2020, the number of new patients with HAE ranged from 3 to 15%. Throughout the calendar years, the prevalence of patients with HAE ranged from 280 patients in 2012 to 509 patients in 2015 with a mean of 401 patients per year.

**Table 2:** New cases and prevalence of HAE and treatment performed by patients with HAE by calendar year (*n* = 799).

	Total	2011	2012	2013	2014	2015	2016	2017	2018	2019	2020	2021
HAE cases, *N* (%)												
New cases*	799 (100%)	281 (35.2%)	74 (9.3%)	116 (14.5%)	93 (11.6%)	50 (6.3%)	33 (4.1%)	24 (3.0%)	57 (7.1%)	38 (4.8%)	22 (2.8%)	11 (1.4%)
Prevalent cases**	777*	–	280	345	439	509	480	446	437	417	347	313
Treatment patterns, *N* (%)												
Prophylactic treatment^1^	799 (100%)	281 (74.1%)	308 (70.0%)	378 (78.3%)	404 (78.9%)	398 (78.7%)	245 (60.2%)	156 (43.2%)	211 (51.1%)	212 (55.6%)	140 (48.3%)	146 (45.3%)
On-demand treatment^2^	20 (2.50%)	1 (0.3%)	4 (0.9%)	1 (0.2%)	3 (0.6%)	1 (0.2%)	4 (0.9%)	3 (0.8%)	3 (0.7%)	2 (0.5%)	2 (0.7%)	1 (0.3%)

* Included patients with the index date in the respective year; ** Patients with more than one year of claim.

1 Within SUS, the only prophylactic treatment available is danazol.

2 Within SUS, the only on-demand treatment available is frozen plasma transfusion.

Over the calendar year, there was a decreasing trend in the proportion of patients treated with danazol, ranging from approximately 74% in 2011 to 45% in 2021. The only on-demand HAE treatment available within SUS is frozen plasma, and 2.5% of the study sample had a record of at least one claim of on-demand treatment during the study. The proportion of patients with on-demand approach ranged from 0.3% in 2011 to 0.3% in 2021, with 2012 and 2016 (0.9%) the years with the higher proportion of patients with records of fresh plasma transfusion (Table 3).

**Table 3:** Treatment for HAE patients.

	Total	2011	2012	2013	2014	2015	2016	2017	2018	2019	2020	2021
Prophylactic treatment												
Unique patient—*n* (%)	799 (100%)	281 (74.14%)	308 (70%)	378 (78.26%)	404 (78.91%)	398 (78.66%)	245 (60.2%)	156 (43.21%)	211 (51.09%)	212 (55.64%)	140 (48.28%)	146 (45.34%)
Number of claims—*n*	23,367	2,669	3,032	3,717	3,912	2,899	1,382	878	1,448	1,545	749	1,136
Claims/patients—ratio	29.25	9.50	9.84	9.83	9.68	7.28	5.64	5.63	6.86	7.29	5.35	7.78
On-demand treatment—*n* (%)												
Unique patient—*n* (%)	20 (2.5%)	1 (0.26%)	4 (0.91%)	1 (0.21%)	3 (0.59%)	1 (0.2%)	4 (0.98%)	3 (0.83%)	3 (0.73%)	2 (0.52%)	2 (0.69%)	1 (0.31%)
Number of claims—*n*	53	2	13	3	11	3	4	4	6	2	4	1
Claims/patients—ratio	2.65	2.00	3.25	3.00	3.67	3.00	1.00	1.33	2.00	1.00	2.00	1.00

Prophylactic treatment: number and proportion of patients with a record of danazol treatment in the study period.

On-demand (acute) treatment: number and proportion of patients with a record of plasma transfusion in the study period.

Table 3 describes the treatment characteristics of HAE patients across the years (2011–2021). There were, in total, 23,367 unique claims of prophylactic treatment that gives for each HAE patient, on average, 29.25 claims across the study period. Between 2011 and 2021, the average number of claims per HAE patient ranged from 5.35 in 2020 to 9.84 in 2012. Regarding HAE patients who received on-demand treatment, there were 53 instances of such treatment during the study period with an average of 2.65 claims per patient. Between 2011 and 2021, the average number of on-demand treatment claims ranged from 1 to 3.67 in 2014.

From the 799 patients identified as HAE and treated with danazol from 2011 to 2021, 253 (32%) patients had a record of at least one attack (report of ICD-10 code and/or procedure pre-defined as attack) over the study period. Of those, 123 (49%) patients had one attack, 42 (17%) had 2 attacks, 30 (12%) patients had 3 attacks, 15 (6%) had 4 attacks, 5 (2%) patients had 5 attacks, 8 (3%) patients had 6 attacks, 4 (2%) patients had 7 attacks, and 26 (10%) patients had 8 or more attacks over the study period. Thus, the proportion of patients with attacks decreased as the number of attacks increased, except for patients with 8 or more attacks. The distribution of patients with attacks per year was approximately 32% across the calendar years considering the population with attack (*n* = 253) and approximately 12% considering the entire HAE population (*n* = 799) with attendance in the respective year (Table 4).

**Table 4:** Proportion of patients with attacks (report of ICD-10 and/or procedures claim defined as a proxy of attack*) by calendar year (*n* = 799).

	Total	2011	2012	2013	2014	2015	2016	2017	2018	2019	2020	2021
Total population with attendance by year	799	379	440	483	512	506	407	361	413	381	290	322
Patients with attack in the respective year, among the total population attended per year—*n* (%)	253 (31.66%)	44 (11.61%)	54 (12.27%)	45 (9.32%)	59 (11.52%)	59 (11.66%)	55 (13.51%)	51 (14.12%)	47 (11.38%)	48 (12.60%)	35 (12.07%)	33 (10.25%)
Attack population with attendance by year (*n*)	253	145	161	167	178	172	151	131	142	138	123	125
Patients with attack in the respective year, among the attack population attended per year—*n* (%)	253 (100%)	44 (30.34%)	54 (33.54%)	45 (26.94%)	59 (33.14%)	59 (34.30%)	55 (36.42%)	51 (38.93%)	47 (33.09%)	48 (34.78%)	35 (28.45%)	33 (26.40%)
Patients with 1 attack	123 (48.6%)	33 (22.8%)	33 (20.5%)	27 (16.2%)	45 (25.3%)	40 (23.3%)	40 (26.5%)	32 (24.4%)	32 (22.5%)	30 (21.7%)	23 (18.7%)	24 (19.2%)
Patients with 2 attacks	42 (16.6%)	8 (5.5%)	14 (8.7%)	12 (7.2%)	8 (4.5%)	12 (7.0%)	7 (4.6%)	10 (7.6%)	9 (6.3%)	8 (5.8%)	5 (4.1%)	3 (2.4%)
Patients with 3 attacks	30 (11.9%)	0 (0.0%)	2 (1.2%)	4 (2.4%)	4 (2.3%)	1 (0.6%)	3 (2.0%)	5 (3.8%)	3 (2.1%)	6 (4.4%)	3 (2.4%)	3 (2.4%)
Patients with 4 attacks	15 (5.9%)	1 (0.7%)	2 (1.2%)	1 (0.6%)	0 (0.0%)	2 (1.2%)	2 (1.3%)	2 (1.5%)	1 (0.7%)	3 (2.2%)	0 (0.0%)	1 (0.8%)
Patients with 5 attacks	5 (2.0%)	1 (0.7%)	1 (0.6%)	0 (0.0%)	1 (0.6%)	1 (0.6%)	1 (0.7%)	0 (0.0%)	1 (0.7%)	0 (0.0%)	2 (1.6%)	0 (0.0%)
Patients with 6 attacks	8 (3.2%)	1 (0.7%)	1 (0.6%)	1 (0.6%)	1 (0.6%)	2 (1.2%)	1 (0.7%)	1 (0.8%)	0 (0.0%)	0 (0.0%)	2 (1.6%)	2 (1.6%)
Patients with 7 attacks	4 (1.6%)	0 (0.0%)	0 (0.0%)	0 (0.0%)	0 (0.0%)	0 (0.0%)	0 (0.0%)	0 (0.0%)	0 (0.0%)	1 (0.7%)	0 (0.0%)	0 (0.0%)
Patients with 8 or more attacks	26 (10.3%)	0(0.0%)	1 (0.6%)	0 (0.0%)	0 (0.0%)	1 (0.6%)	1 (0.7%)	1 (0.8%)	1 (0.7%)	0 (0.0%)	0 (0.0%)	0 (0.0%)
Hospitalizations with ICD-10 D84.1 code—*n* (%)**	1 (0.4%)	0 (0.0%)	0 (0.0%)	0 (0.0%)	0 (0.0%)	0 (0.0%)	0 (0.0%)	1 (2.0%)	0 (0.0%)	0 (0.0%)	0 (0.0%)	0 (0.0%)
Procedures—*n* of patients (%)												
General hospitalization	150 (59.3%)	31 (21.4%)	36 (22.4%)	41 (24.6%)	46 (25.8%)	47 (27.3%)	52 (34.4%)	48 (36.6%)	50 (35.2%)	51 (36.9%)	48 (39.0%)	36 (28.8%)
Procedure HAE-related hospitalization	45 (17.8%)	2 (1.4%)	3 (1.9%)	2 (1.2%)	7 (3.9%)	5 (2.9%)	3 (2.0%)	8 (6.1%)	5 (3.5%)	7 (5.1%)	6 (4.9%)	5 (4.0%)
ICD-10 HAE-related hospitalization	128 (50.6%)	13 (8.9%)	19 (11.8%)	24 (14.4%)	20 (11.2%)	36 (20.9%)	27 (17.9%)	34 (25.9%)	29 (20.4%)	31 (22.5%)	26 (21.1%)	26 (20.8%)
ICU hospitalization	77 (30.4%)	12 (8.3%)	14 (8.7%)	20 (12.0%)	24 (13.5%)	20 (11.6%)	20 (13.3%)	18 (13.7%)	16 (11.3%)	18 (13.0%)	17 (13.8%)	13 (10.4%)
ICD-10 HAE-related ICU	25 (9.9%)	0 (0.0%)	1 (0.6%)	3 (1.8%)	2 (1.1%)	4 (2.3%)	3 (2.0%)	7 (5.3%)	1 (0.7%)	4 (2.9%)	3 (2.4%)	4 (3.2%)
Treatment of other diseases of the digestive tract	24 (9.5%)	2 (4.6%)	1 (1.9%)	1 (2.2%)	5 (8.5%)	3 (5.1%)	3 (5.5%)	2 (3.9%)	4 (8.5%)	0 (0.0%)	1 (2.9%)	4 (12.1%)
Plasma transfusion	20 (7.9%)	1 (2.3%)	4 (7.4%)	1 (2.2%)	3 (5.1%)	1 (1.7%)	4 (7.3%)	3 (5.9%)	3 (6.4%)	2 (4.2%)	2 (5.7%)	1 (3.0%)
Anaphylactic shock treatment	12 (4.7%)	1 (2.3%)	2 (3.7%)	1 (2.2%)	1 (1.7%)	0 (0.0%)	0 (0.0%)	2 (3.9%)	1 (2.1%)	3 (6.3%)	1 (2.9%)	1 (3.0%)
Treatment of other bowel diseases	8 (3.2%)	0 (0.0%)	0 (0.0%)	0 (0.0%)	1 (1.7%)	0 (0.0%)	0 (0.0%)	1 (1.9%)	1 (2.1%)	3 (6.3%)	2 (5.7%)	0 (0.0%)
Tracheostomy	7 (2.8%)	2 (4.6%)	2 (3.7%)	1 (2.2%)	1 (1.7%)	0 (0.0%)	0 (0.0%)	0 (0.0%)	0 (0.0%)	0 (0.0%)	1 (2.9%)	0 (0.0%)
Treatment of other respiratory tract diseases	6 (2.4%)	0 (0.0%)	0 (0.0%)	0 (0.0%)	1 (1.7%)	2 (3.4%)	0 (0.0%)	2 (3.9%)	0 (0.0%)	1 (2.1%)	1 (2.9%)	0 (0.0%)
Physiotherapeutic care in patients with non-systemic respiratory disorder	5 (2.0%)	1 (2.3%)	1 (1.9%)	0 (0.0%)	1 (1.7%)	2 (3.4%)	1 (1.8%)	1 (1.9%)	0 (0.0%)	0 (0.0%)	0 (0.0%)	2 (6.1%)
Treatment of other upper respiratory diseases	3 (1.2%)	0 (0.0%)	0 (0.0%)	0 (0.0%)	0 (0.0%)	0 (0.0%)	1 (1.8%)	2 (3.9%)	0 (0.0%)	0 (0.0%)	0 (0.0%)	0 (0.0%)
Treatment of enteritis and non-infectious colitis	1 (0.4%)	0 (0.0%)	0 (0.0%)	1 (2.2%)	0 (0.0%)	0 (0.0%)	0 (0.0%)	0 (0.0%)	0 (0.0%)	0 (0.0%)	0 (0.0%)	0 (0.0%)
Physiotherapeutic care in patients with a systemic respiratory disorder	1 (0.4%)	0 (0.0%)	0 (0.0%)	0 (0.0%)	0 (0.0%)	0 (0.0%)	0 (0.0%)	0 (0.0%)	1 (2.1%)	0 (0.0%)	0 (0.0%)	0 (0.0%)
Treatment—*n* (%)												
Plasma transfusion	20 (7.9%)	1 (2.3%)	4 (7.4%)	1 (2.2%)	3 (5.1%)	1 (1.7%)	4 (7.3%)	3 (5.9%)	3 (6.4%)	2 (4.2%)	2 (5.7%)	1 (3.0%)
ICD-10 codes—*n* (%)												
Acute abdomen (abdominal and pelvic pain)	106 (41.9%)	13 (29.6%)	16 (29.6%)	22 (48.9%)	13 (22.0%)	23 (38.9%)	20 (36.4%)	22 (43.1%)	13 (27.7%)	14 (29.2%)	11 (31.4%)	11 (33.3%)
Other and unspecified abdominal pain	67.0 (26.5%)	12 (27.3%)	10 (18.5%)	8 (17.8%)	15 (25.4%)	20 (33.9%)	12 (21.8%)	5 (9.8%)	8 (17.0%)	9 (18.8%)	4 (11.4%)	6 (18.2%)
Other appendicitis	47.0 (18.6%)	4 (9.1%)	13 (24.1%)	9 (20.0%)	12 (20.3%)	12 (20.3%)	6 (10.9%)	9 (17.7%)	14 (29.8%)	10 (20.8%)	13 (37.1%)	9 (27.3%)
Angioneurotic edema	27.0 (10.7%)	1 (2.3%)	6 (11.1%)	2 (4.4%)	2 (3.4%)	1 (1.7%)	5 (9.1%)	4 (7.8%)	3 (6.4%)	7 (14.6%)	2 (5.7%)	1 (3.0%)
Pain, unspecified	16.0 (6.3%)	6 (13.6%)	7 (12.9%)	2 (4.4%)	0 (0.0%)	2 (3.4%)	3 (5.5%)	1 (1.96%)	1 (2.1%)	4 (8.3%)	0 (0.0%)	1 (3.0%)
Edema, unspecified	13.0 (5.1%)	0 (0.0%)	2 (3.7%)	2 (4.4%)	5 (8.5%)	2 (3.4%)	0 (0.0%)	3 (5.9%)	1 (2.1%)	0 (0.0%)	2 (5.7%)	0 (0.0%)
Acute tonsillitis, unspecified	13.0 (5.1%)	2 (4.6%)	0 (0.0%)	1 (2.2%)	0 (0.0%)	1 (1.7%)	2 (3.6%)	2 (3.9%)	2 (4.3%)	2 (4.2%)	1 (2.9%)	0 (0.0%)
Acute pain	12.0 (4.7%)	2 (4.6%)	2 (3.7%)	1 (2.2%)	2 (3.4%)	1 (1.7%)	2 (3.6%)	0 (0.0%)	1 (2.1%)	1 (2.1%)	1 (2.9%)	1 (3.0%)
Anaphylactic shock, unspecified	7.0 (2.8%)	0 (0.0%)	1 (1.9%)	1 (2.2%)	0 (0.0%)	0 (0.0%)	0 (0.0%)	2 (3.9%)	1 (2.1%)	1 (2.1%)	1 (2.9%)	0 (0.0%)
Other chronic pain	6.0 (2.4%)	1 (2.3%)	2 (3.7%)	0 (0.0%)	1 (1.7%)	0 (0.0%)	1 (1.8%)	1 (1.9%)	1 (2.1%)	2 (4.2%)	0 (0.0%)	1 (3.0%)
Acute respiratory failure	4.0 (1.6%)	0 (0.0%)	1 (1.9%)	0 (0.0%)	0 (0.0%)	1 (1.7%)	0 (0.0%)	2 (3.9%)	0 (0.0%)	1 (2.1%)	0 (0.0%)	0 (0.0%)
Non-specific respiratory disorders	4.0 (1.6%)	1 (2.3%)	0 (0.0%)	0 (0.0%)	1 (1.7%)	0 (0.0%)	0 (0.0%)	0 (0.0%)	1 (2.1%)	0 (0.0%)	0 (0.0%)	1 (3.0%)
Unspecified appendicitis	3.0 (1.2%)	0 (0.0%)	0 (0.0%)	0 (0.0%)	0 (0.0%)	2 (3.4%)	1 (1.8%)	1 (1.9%)	0 (0.0%)	0 (0.0%)	0 (0.0%)	0 (0.0%)
Acute respiratory failure, unspecified	3.0 (1.2%)	0 (0.0%)	1 (1.9%)	1 (2.2%)	1 (1.7%)	0 (0.0%)	0 (0.0%)	0 (0.0%)	0 (0.0%)	0 (0.0%)	0 (0.0%)	0 (0.0%)
Allergy, unspecified	3.0 (1.2%)	1 (2.3%)	0 (0.0%)	0 (0.0%)	1 (1.7%)	0 (0.0%)	1 (1.8%)	0 (0.0%)	0 (0.0%)	0 (0.0%)	0 (0.0%)	1 (3.0%)
Edema of the larynx	3.0 (1.2%)	1 (2.3%)	0 (0.0%)	0 (0.0%)	0 (0.0%)	0 (0.0%)	1 (1.8%)	0 (0.0%)	1 (2.1%)	0 (0.0%)	0 (0.0%)	0 (0.0%)
Abdominal pain	2.0 (0.8%)	0 (0.0%)	0 (0.0%)	0 (0.0%)	0 (0.0%)	0 (0.0%)	0 (0.0%)	0 (0.0%)	0 (0.0%)	0 (0.0%)	0 (0.0%)	2 (6.1%)
Stridor	2.0 (0.8%)	0 (0.0%)	0 (0.0%)	0 (0.0%)	0 (0.0%)	1 (1.7%)	0 (0.0%)	0 (0.0%)	0 (0.0%)	1 (2.1%)	0 (0.0%)	0 (0.0%)
Breathlessness	1.0 (0.4%)	0 (0.0%)	0 (0.0%)	0 (0.0%)	0 (0.0%)	1 (1.7%)	0 (0.0%)	0 (0.0%)	0 (0.0%)	0 (0.0%)	0 (0.0%)	0 (0.0%)

*Pre-defined as attack in the protocol.

**Patients with a record of ICD-10 D84.1 hospitalization.

### 3.3 Hospital admissions after HAE attack

Of the 253 patients with a record of at least one HAE attack over the study period, 150 (59.3%) had at least one hospital admission by any cause, 45 (17.8%) patients had at least one hospital admission with HAE-related procedures, and 128 (50.6%) had at least one hospital admission with HAE-related ICD-10 codes. In total, 77 (30.4%) patients had a record of at least one intensive care unit (ICU) admission by any cause, and 25 (9.9%) patients had HAE-related ICU admissions. Most frequent procedures reported as HAE attacks were treatment of other diseases of the digestive tract (9.5%), plasma transfusion (7.9%), anaphylactic shock treatment (4.7%), treatment of other bowel diseases (3.2%), and tracheostomy (2.8%). The ICD-10 codes most reported as HAE attack were acute abdomen (41.9%), other and unspecified abdominal pain (26.5%), other appendicitis (18.6%), angioneurotic edema (10.7%), and pain unspecified (6.3%) (Table 4).

The distribution of patients with attack per year (1st to 11th year) after danazol initiation is described in Table 5. Overall, in the first year after danazol initiation, the proportion of patients with attacks is 12.5%. Over the years, the proportion of patients with attacks, even though using danazol, remained between 8.6 and 15.8%. The proportion of patients with 1 attack ranged from 67 to 33% and the proportion of patients with 2 attacks ranged from 19 to 33% in the first year to the last year (11 years), respectively. Other unspecified abdominal pain, acute abdominal, and other appendicitis were the most reported ICD-10 codes across all the years after the index date. The most reported procedures across all the years were plasma transfusion, treatment of other diseases of the digestive tract, and tracheostomy (Table 5).

**Table 5:** Proportion of patients with attacks (report of ICD-10 and/or procedures claim defined as a proxy of attack*) by year after the index date (*n* = 799).

	1 year	2 year	3 year	4 year	5 year	6 year	7 year	8 year	9 year	10 year	11 year
	*n* = 799	*n* = 577	*n* = 465	*n* = 385	*n* = 332	*n* = 251	*n* = 220	*n* = 183	*n* = 120	*n* = 95	*n* = 35
Patients with attack in the respective year—*n* (%)	100 (12.5%)	63 (10.9%)	60 (12.9%)	48 (12.5%)	35 (10.5%)	33 (13.1%)	29 (13.2%)	26 (14.2%)	12 (10.0%)	15 (15.8%)	3 (8.6%)
Patients with 1 attack	67 (67.0%)	44 (69.8%)	46 (76.7%)	35 (72.9%)	27 (77.1%)	24 (72.7%)	24 (82.8%)	14 (53.8%)	3 (25.0%)	10 (66.7%)	1 (33.3%)
Patients with 2 attacks	19 (19.0%)	9 (14.3%)	7 (11.7%)	6 (12.5%)	3 (8.6%)	5 (15.2%)	2 (6.9%)	4 (15.4%)	6 (50.0%)	1 (6.7%)	1 (33.3%)
Patients with 3 attacks	5 (5.0%)	3 (4.8%)	4 (6.7%)	4 (8.3%)	2 (5.7%)	1 (3.0%)	1 (3.4%)	3 (11.5%)	0 (0.0%)	2 (13.3%)	1 (33.3%)
Patients with 4 attacks	2 (2.0%)	4 (6.3%)	0 (0.0%)	2 (4.2%)	2 (5.7%)	3 (9.1%)	1 (3.4%)	2 (7.7%)	0 (0.0%)	0 (0.0%)	0 (0.0%)
Patients with 5 attacks	3 (3.0%)	0 (0.0%)	1 (1.7%)	0 (0.0%)	1 (2.9%)	0 (0.0%)	0 (0.0%)	2 (7.7%)	2 (16.7%)	1 (6.7%)	0 (0.0%)
Patients with 6 attacks	2 (2.0%)	3 (4.8%)	1 (1.7%)	1 (2.1%)	0 (0.0%)	0 (0.0%)	0 (0.0%)	1 (3.8%)	0 (0.0%)	1 (6.7%)	0 (0.0%)
Patients with 7 attacks	1 (1.0%)	0 (0.0%)	0 (0.0%)	0 (0.0%)	0 (0.0%)	0 (0.0%)	0 (0.0%)	0 (0.0%)	1 (8.3%)	0 (0.0%)	0 (0.0%)
Patients with 8 or more attacks	1 (1.0%)	0 (0.0%)	1 (1.7%)	0 (0.0%)	0 (0.0%)	0 (0.0%)	1 (3.4%)	0 (0.0%)	0 (0.0%)	0 (0.0%)	0 (0.0%)

*Pre-defined as attack in the protocol.

**Figure 3:**
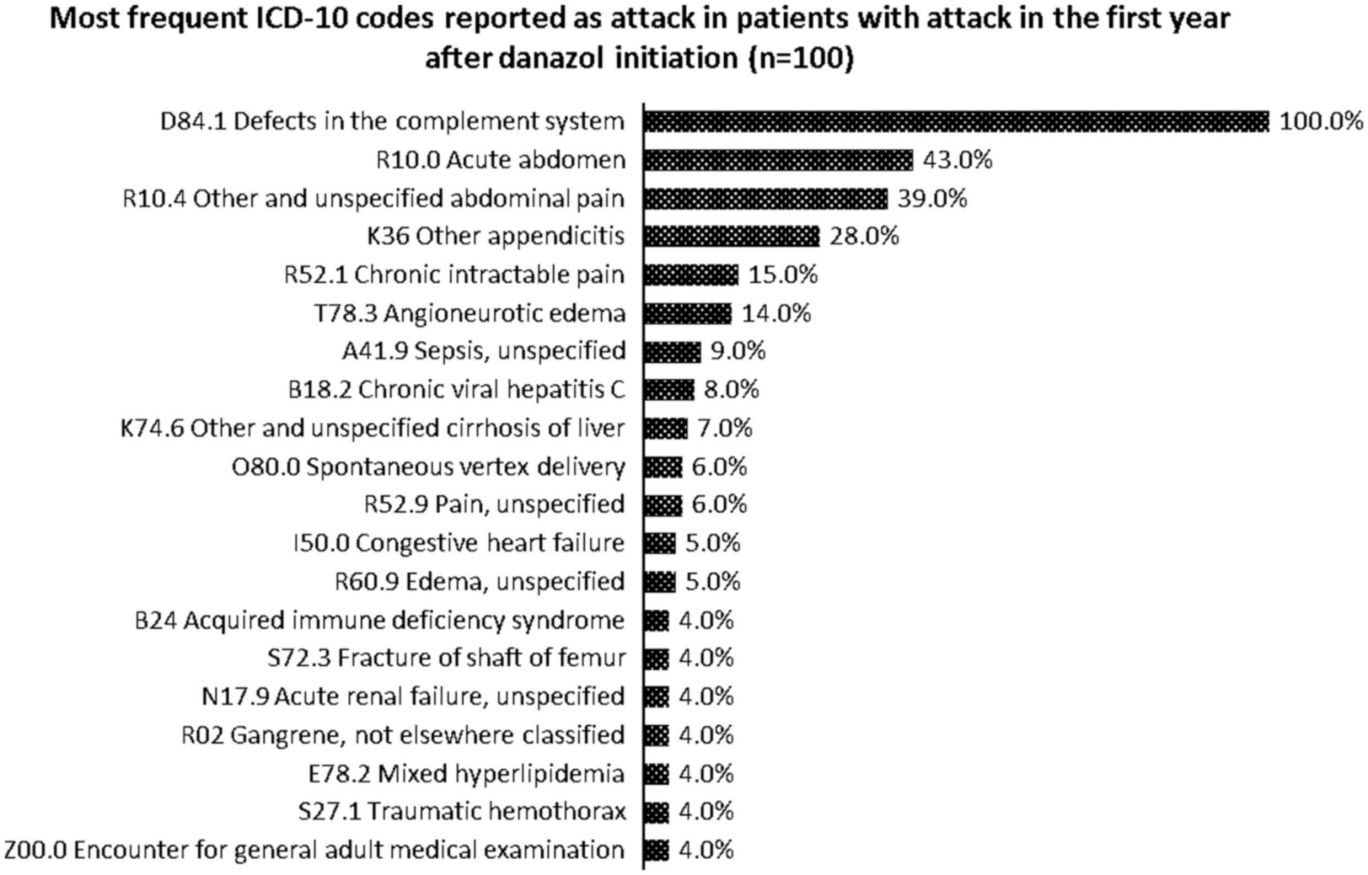
Most frequent ICD-10 codes reported as attack in patients with attack in the first year after danazol initiation (*n* = 100).

Most reported ICD-10 codes were D84.1—defects in the complement system, R10.0—acute abdomen, R10.4—other and unspecified abdominal pain, K36—other appendicitis, and R52.1—chronic intractable pain. Figure 3 describes the top-20 ICD-10 codes reported by the patients who had attacks in the first year of danazol initiation (*n* = 100).

Table 6 describes patients with severe attack during the study period (total), and Supplementary Table S5 describes patients with severe attack per calendar year (2011–2021). Of the 799 patients in the HAE cohort, 29 (3.6%) patients had a record of at least one severe attack (report of ICD-10 code and/or procedure pre-defined) over the study period. Most patients (76%) had only one severe attack, 7% had 2 or more severe attacks, 4% had 6 severe attacks, 4% had 7 severe attacks, and 7% had 8 or more severe attacks. Thus, the distribution of patients with severe attacks decreased proportionally as the number of attacks per patient increased, except for patients with 8 or more attacks. The distribution of patients with severe attacks per calendar year when considering all HAE population in that given year ranged from 0.41% in 2013 to 1.14% in 2014 (Supplementary Table S5).

**Table 6:** Proportion of patients with severe attacks (report of ICD-10 and/or procedures claim defined as a proxy of severe attack*) by calendar year (*n* = 799).

	Total
Total population with attendance by year	*n* = 799
Patients with severe attack in the respective year, among the total population attended per year—*n* (%)	29 (3.6%)
Severe attack population with attendance by year	29
Patients with severe attack in the respective year—*n* (%)	29 (100%)
Patients with 1 attack	22 (75.9%)
Patients with 2 attacks	2 (6.9%)
Patients with 3 attacks	1 (3.5%)
Patients with 4 attacks	0 (0.0%)
Patients with 5 attacks	0 (0.0%)
Patients with 6 attacks	1 (3.5%)
Patients with 7 attacks	1 (3.5%)
Patients with 8 or more attacks	2 (6.9%)
General hospitalization—*n* of patients (%)	22 (75.9%)
General ICU hospitalization—*n* of patients (%)	13 (44.8%)
ICD-10 HAE-related ICU—*n* of patients (%)	2 (6.9%)
Anaphylactic shock treatment	12 (41.0%)
Tracheostomy	7 (24.0%)
Physiotherapeutic care in patients with non-systemic respiratory disorder	5 (17.0%)
Physiotherapeutic care in patients with a systemic respiratory disorder	1 (3.0%)
ICD-10 HAE-related hospitalization—*n* of patients (%)	9 (31.0%)
Anaphylactic shock, unspecified	7 (28.0%)
Breathlessness	1 (4.0%)
Edema of the larynx	1 (4.0%)

*Severe attack: list of procedures and ICD-10 codes presented in Supplementary Table IV.

For those 29 patients with severe attacks over the study period, a total of 22 (76%) patients had a least one hospital admission by any cause, 9 (31%) patients had a hospital admission reporting an ICD-10 code pre-defined as severe attack, 13 (45%) patients had an ICU admission by any cause, and 2 (7%) patients had an ICU admission reporting ICD-10 codes pre-defined as severe attack. The distribution of patients with severe attack that were hospitalized (any cause or HAE-related) increased over the years. Overall, the anaphylactic shock treatment and tracheostomy were the most frequent procedures recorded for the patients with severe attack over the study period. Anaphylactic shock, breathlessness, and edema of the larynx were the most frequent ICD-10 codes reported for the patient with severe attack over the study period (Table 6).

Table 7 describes the HAE-related healthcare resource usage from the HAE patients with attacks (*n* = 253). From the 253 patients with a record of at least one attack during the study period, 491 admissions in hospital were recorded. The mean (SD) hospitalizations per patient were 3 (5) and 2 (2). The median (IQR) hospitalizations per patient were 0.66 (0.95) and 0 (1.1) hospitalizations PPPY. Almost 55% (*n* = 137) of patients with attack had at least one HAE-related hospital admission (with record of ICD-10 code and/or procedure related to HAE). For those patients with HAE-related hospitalization (*n* = 137), 95 (69%) patients had 1 to 2 hospital admissions, 22 (16%) patients had 3 to 6 hospital admissions, and 20 (15%) patients had 6+ hospital admissions.

**Table 7:** HAE-related healthcare resource utilization of HAE patients who had attacks over the study period (*n* = 253).

Healthcare resource utilization	HAE
	*n* = 253
**Inpatient setting**	
Hospital admissions (*n*)	491
Mean (SD) per patient	3 (5)
Median (IQR) per patient	2 (2)
Mean (SD) per patient per year (PPPY)	0.66 (0.95)
Median (IQR) per patient per year (PPPY)	0 (1.1)
Patients with at least one admission, *n* (%)	137 (54.15%)
Patients with 1 to 2 admissions, *n* (%)	95 (69.34%)
Patients with 3 to 6 admissions, *n* (%)	22 (16.06%)
Patients with 6+ admissions, *n* (%)	20 (14.6%)
Total length of stay (days)	
Mean (SD)	5 (8)
Median (IQR)	3 (5)
ICU admissions (*n*)	57
Mean (SD) per patient	1 (1)
Median (IQR) per patient	1 (0)
Mean (SD) per patient per year (PPPY)	0.25 (0.61)
Median (IQR) per patient per year (PPPY)	0 (0)
Patients with at least one ICU admission, *n* (%)	36 (14.23%)
Patients with 1 to 2 admissions, *n* (%)	32 (88.89%)
Patients with 3 to 6 admissions, *n* (%)	3 (8.33%)
Patients with 6+ admissions, *n* (%)	1 (2.78%)
**Outpatient setting**	
Outpatient visits (*n*)	188
Mean (SD) per patient	2 (2)
Median (IQR) per patient	1 (2)
Mean (SD) per patient per year (PPPY)	0.57 (1.45)
Median (IQR) per patient per year (PPPY)	0 (0.54)
Patients with at least one outpatient visit, *n* (%)	79 (31.23%)

Only outpatient or inpatient visit after index date were considered for the healthcare resource utilization.

HAE related to admissions in outpatients, hospital, and ICU.

The mean (SD) hospitalization length of stay was 5 (8) days. Over 14% (*n* = 36) of HAE patients with attack (*n* = 253) had at least one HAE-related ICU admission. Of those patients with at least one ICU admission (*n* = 36), 89% had 1 to 2 ICU admissions, 8% had 3 to 6 ICU admissions, and 3% had 6+ ICU admissions. For the outpatient setting, a total of 188 HAE-related outpatient visits were performed by the 253 patients with attack. The mean (SD) numbers of outpatient visits per patient were 2 (2) and 1 (2). The median (IQR) numbers of outpatient visits per patient were 0.57 (1.45) and 0 (0.54) visits PPPY. Only 79 (31%) of the HAE patients with attack record of HAE-related outpatient visits (Table 7).

The HAE-related HCRU per each year after the index date is described in Table 8. In the first year after the index date, patients who had attacks had 79 HAE-related hospital admissions, of which 50 (20%) had at least one HAE-related hospitalization. For those patients with at least one hospital admission (*n* = 50), almost 88% of them had 1 to 2 hospitalizations, 12% had 3 to 6 hospitalizations, and none had 6+ HAE-related hospitalizations. The absolute number of patients with any hospital admission decreased over the years. The mean hospitalization length of stay in hospital was 5 (7) days in the first year after the index date (Table 8).

**Table 8:** HAE-related healthcare resource utilization (inpatient and outpatient) of patients with HAE that had attack over the study period (*n* = 253) per year after the index date.

	1 year	2 year	3 year	4 year	5 year	6 year	7 year	8 year	9 year	10 year	11 year
	*n* = 253	*n* = 196	*n* = 170	*n* = 144	*n* = 126	*n* = 93	*n* = 84	*n* = 73	*n* = 46	*n* = 43	*n* = 13
**Inpatient setting**											
Hospital admissions (*n*)	79	63	67	59	43	37	33	49	27	28	6
Mean (SD) per patient*	1 (1)	1 (0)	1 (1)	1 (1)	1 (0)	1 (1)	1 (0)	2 (1)	2 (1)	2 (1)	2 (1)
Median (IQR) per patient*	1 (1)	1 (1)	1 (1)	1 (0)	1 (0)	1 (1)	1 (0)	1 (2)	2 (0)	1 (1)	2 (1)
Patients with at least one hospital admission, *n* (%)	50 (19.76%)	43 (21.94%)	42 (24.71%)	39 (27.08%)	30 (23.81%)	23 (24.73%)	26 (30.95%)	23 (31.51%)	11 (23.91%)	14 (32.56%)	3 (23.08%)
Patients with 1 to 2 admissions, *n* (%)	44 (88%)	38 (88.37%)	36 (85.71%)	33 (84.62%)	26 (86.67%)	20 (86.96%)	24 (92.31%)	16 (69.57%)	9 (81.82%)	10 (71.43%)	2 (66.67%)
Patients with 3 to 6 admissions, *n* (%)	6 (12%)	5 (11.63%)	5 (11.9%)	6 (15.38%)	4 (13.33%)	3 (13.04%)	2 (7.69%)	7 (30.43%)	1 (9.09%)	4 (28.57%)	1 (33.33%)
Patients with 6+ admissions, *n* (%)	0 (0%)	0 (0%)	1 (2.38%)	0 (0%)	0 (0%)	0 (0%)	0 (0%)	0 (0%)	1 (9.09%)	0 (0%)	0 (0%)
Total length of stay (days)											
Mean (SD)	5 (7)	4 (5)	7 (16)	4 (5)	5 (6)	5 (5)	4 (6)	3 (3)	5 (5)	3 (3)	1 (1)
Median (IQR)	3 (4.5)	2 (5)	3 (4)	3 (4)	3 (5)	4 (4)	2 (3)	3 (4)	3 (4.5)	2 (3)	1 (1)
ICU admissions (*n*)	6	4	20	8	2	4	5	2	3	2	1
Mean (SD) per patient*	1 (0)	1 (0)	1(2)	1 (0)	1 (0)	1 (0)	1 (0)	1 (0)	1 (0)	1 (0)	1 (0)
Median (IQR) per patient*	1 (0)	1 (0)	1(0)	1 (0)	1 (0)	1 (0)	1 (0)	1 (0)	1 (0)	1 (0)	1 (0)
Patients with at least one ICU admission, *n* (%)	5 (1.98%)	4 (2.04%)	11 (6.47%)	8 (5.56%)	2 (1.59%)	4 (4.3%)	5 (5.95%)	2 (2.74%)	3 (6.52%)	2 (4.65%)	1 (7.69%)
Patients with 1 to 2 admissions, *n* (%)	5 (100%)	4 (100%)	9 (81.82%)	8 (100%)	2 (100%)	4 (100%)	5 (100%)	2 (100%)	3 (100%)	2 (100%)	1 (100%)
Patients with 3 to 6 admissions, *n* (%)	0 (0%)	0 (0%)	1 (9.09%)	0 (0%)	0 (0%)	0 (0%)	0 (0%)	0 (0%)	0 (0%)	0 (0%)	0 (0%)
Patients with 6+ admissions, *n* (%)	0 (0%)	0 (0%)	1 (9.09%)	0 (0%)	0 (0%)	0 (0%)	0 (0%)	0 (0%)	0 (0%)	0 (0%)	0 (0%)
**Outpatient setting**											
Total number of outpatient visits (*n*)	59	42	25	14	9	12	15	6	5	1	0
Mean (SD) per patient*	1 (1)	1 (1)	1 (1)	1 (1)	1 (1)	1 (0)	5 (6)	2 (1)	5 (−)	1 (−)	0
Median (IQR) per patient*	1 (0)	1 (0)	1 (0)	1 (1)	1 (0)	1 (0)	1 (6)	2 (1)	5 (−)	1 (−)	0
Patients with at least one outpatient visit, *n* (%)	38 (15.02%)	23 (11.73%)	19 (11.18%)	9 (6.25%)	5 (3.97%)	10 (10.75%)	3 (3.57%)	3 (4.11%)	1 (2.17%)	1 (2.33%)	0 (0%)

Healthcare resource use after the index date: first claim of danazol.

Inpatient setting: included all general admissions in hospital and ICU after the index date.

*Mean/median per patient: Total of hospital/intensive care/outpatient admissions per patient divided by total time number of patients.

Considering the intensive care admissions, it was identified that almost 2% of the patients had at least one HAE-related ICU admission in the first year after danazol initiation, and 2, 6.5, 5.6, 1.6, 4.3, 6.0, 2.7, 6.5, 4.7, and 7.7% had at least 1 HAE-related ICU admission in the following years (2- to 11-year post-index). All patients with ICU admission HAE-related had between 1 and 2 ICU admissions after the index date (Table 8).

From the outpatient perspective, a total of 59 HAE-related visits were performed by the 38 patients who had at least one visit in the first year, followed by 42 visits performed by 23 patients who had at least one visit in the second year and 25 visits performed by 19 patients who had at least one visit in the third year after danazol initiation. An average, 1 outpatient HAE-related visit was performed in the first year after danazol initiation, and the trend follows for the following years (Table 8).

## 4 Discussion

This database study of the ultra-rare disease, HAE, conducted from January 2011 until December 2021 is the first of its kind to describe the characteristics of patients with HAE-C1-INH treated with danazol within the SUS and yielded interesting findings. During the follow-up period, our study included 799 patients with a mean age at danazol initiation of 40 years (16). The number of patients with HAE showed a similar distribution over these 10-year period analyzed with the highest number of patients in 2015 (*n* = 509) and 2016 (*n* = 480). The prevalence of patients with HAE throughout the calendar years showed a similar distribution and the mean prevalence was approximately 401 patients. Approximately 12% of the patients included in the study had at least one attack per year. In addition, from those patients who had attacks, approximately 54% of the patients had at least one hospital admission with HAE-related ICD-10 codes with a mean hospitalization length of 5 (8) days. Interestingly, the number of patients with attacks decreased over the years, while the proportion of patients with any HAE-related hospital admission seems to increase over the years.

The states with the highest concentration of patients identified with HAE and treated with danazol were São Paulo, Minas Gerais, and Paraná. Indeed, these regions reflect one of the country’s highest population densities, but the cases of HAE found seem not equivalent to the demographic density. Probably, the higher number of patients in the Southeast and South regions might be related to the fact these regions concentrated the higher number of specialized centers in the country. According to the ABRANGHE, only 3% of patients registered as HAE were located in the North and less than 8% in the Midwest region of Brazil (20).

The treatment strategy involving prophylaxis drugs to reduce HAE-related morbidity and mortality is mostly related to laryngeal angioedema. This type of attacks can be managed following local anaphylaxis which commonly included intubation or cricothyrotomy/tracheotomy (21). Despite the use of danazol, our study showed that 2.8% of the patients had a tracheostomy and 4.7% of the cases had anaphylactic shock treatment, which is directly related to attacks in upper airways. A Chinese study found that 9 of 43 patients had tracheostomy during an upper airway episode (22). Although the understanding of the pathophysiology of the disease has led to the launch of new treatments (23), in Brazil the access to novel agents, particularly in the public setting, is still restricted. International guidelines preconize the use of C1-INH concentrate and kallikrein inhibitors as the first-line therapy aiming to reduce the frequency and severity of the attacks (17). However, these drugs are not currently available in the Brazilian public setting. Up to now, Danazol is the only drug available in SUS for long-term prophylactic treatment (16), and fresh frozen plasma is used for on-demand treatment predominantly in a hospital setting.

On-demand therapy seems not to be used frequently by HAE treaters (24), in agreement with our results, in which less than 1% of the HAE cohort had at least one claim of fresh frozen plasma over the years. Frozen plasma can be used if specific therapies for HAE-C1-INH are not available. Although fresh frozen plasma treatment can be effective, it is also potentially dangerous to the patient. This is because high-molecular weight kininogen in the presence of other enzymes activated during the attack might raise the levels of bradykinin and worsen the attack (25). Therefore, the study adopts the use of danazol as an eligibility criterion to enhance the accuracy of identifying HAE patients as danazol is the only drug available in the SUS for long-term treatment (16). In addition, the study sought to further understand the impact of danazol use on the frequency of attacks and the utilization of healthcare resources. It is not possible to assume that patients who were treated with danazol continued to use it over the years. However, we found that although there was a decrease in trend in the number of patients with at least one claim of danazol over the years, 43–80% of them were using danazol over the years (Table 1), This allows presuming that most of patients were under prophylactic treatment in the present study, since there is no guarantee that patient would continue the treatment throughout the study period. Adding to this, Table 2 shows the ratio claims for prophylactic and on-demand treatment for patients with HAE. The number of claims of prophylactic treatment (danazol) per patient per year varies from 5.35 to 9.84, and the number of claims per patient per year of on-demand treatment (plasma transfusion) varies from 1 to 3.67 claims per patient, indicating that although there is variation in the number of claims per patient, they are receiving some treatment (Table 2).

The symptoms of HAE are characterized by recurrent episodes of swelling and attacks that are disabling and potentially life-threatening, and are unpredictable in terms of frequency, severity, and local (6). A Brazilian cohort study with 98 HAE patients showed that the main sites affected were extremities (98%), face (88%), abdominal (88%), genitalia (57%), larynx (47%), and tongue (37%) (11). This study corroborates findings as our study showed that attacks were more peripheral (47%), followed by abdominal attacks (39%), facial attacks (6.3%), and laryngeal attacks (4.8%). Episodes involving the intestinal tract cause severe abdominal pain, and as shown by previous studies, approximately 25% of undiagnosed patients might be submitted to an unnecessary surgery, such as abdominal exploratory surgery (13, 14). Symptoms involving upper airway swelling are the most dangerous episode of HAE due to the risk of asphyxia (26) that might affect approximately 25 to 40% of patients not properly treated (11). The present study reported only resources linked to angioedema attacks and the disease itself. Indeed, D84.1 ICD-10 code was not included as attack in the present study to avoid underestimation of attacks since the ICD-10 code was applied as eligibility criteria.

We found that the prevalence of patients who had at least one attack during the period analyzed was approximately 30% and the annual incidence was approximately 25% per each calendar year. In general, we identified 253 patients with at least one attack with most of them (48%) presenting only one attack within the 11-year study period, and among those patients with attacks, approximately 10% had 8 or more attacks per patient. Although almost half of the patients had only one attack, we found a high variability across patients, ranging from 1 to 126 attacks. A multinational post-marketing study of icatibant with 42 patients from Brazil found a total of 228 icatibant-treated attacks over the course of an average of 4 years of follow-up, and a 1-year prospective cost analysis and survival analysis found that in addition to the significant reduction on attack duration after icatibant and pdC1-INH use, an average of 11 attacks per year were reported (27). A survey study conducted on HAE patients in the United States in 2007 showed that patients experienced an average of 27 swelling attacks per year, most of which were of moderate-to-severe intensity (9). The discrepancy in the outcomes could be explained by the inherent limitation on the database used and the proxy considered as attacks in this study. The types of procedures available on SIGTAP are limited, and the lack of clinical characteristics (signs and symptoms) prevents the capture of mild swelling attacks and/or all cases of attacks, which probably underestimated our outcomes. Data from this study were captured from attacks that generated the use of health resources, as recorded by DATASUS. Even with these limitations, it is possible to note the considerable number of attacks presented by HAE patients.

HCRU outcomes of interest were based on hospitalizations, emergency department visits, and ambulatory visits. Although it is known that the disease has an important indirect use of resources and a substantial impact on work production due to work/school absenteeism and productivity impairment during and in between attacks (28), this aspect was not assessed and considered in the present study. HCRU was classified as HAE-related as identified by ICD-10 codes or procedures related to the disease. Among HAE patients treated with danazol with at least one attack (*n* = 253), it was identified that HCRU was higher in hospital settings compared to outpatient settings when considering only resources used to manage attacks. A total of 188 ambulatorial visits and 491 hospital admissions were identified, which represents a mean of 2 and 3 visits per patient over the study period, respectively. Of the 137 patients with at least one hospital admission, nearly 15% had 6 or more hospital admissions and 14% of the patients had at least one ICU admission, demonstrating how serious were the attacks and how much the patients demanded from complex health assistance and hospitalization. The most reported procedures across all the years were plasma transfusion, treatment of other diseases of the digestive tract, and tracheostomy.

A cross-sectional study from a C1-INH-HAE cohort in Brazil from 2010 to 2020 showed that approximately 60% of patients diagnosed with HAE were hospitalized at the *Hospital das Clínicas* of the School of Medicine University of São Paulo, and 30% had an ICU admission (11). An international survey with 242 HAE patients from different countries who have had at least one attack in the last 2 years found that almost all patients (97%) reported regularly seeing a medical professional to manage the disease in the last year, 37% at least one HAE-related emergency room visit, 19% a hospitalization, and 18% an urgent care visit (29). Similarly, in the present study, we found that approximately 20% of the HAE patients who had attacks had at least one HAE-related hospital admission 1 year after danazol initiation. In subsequent years, the proportion of patients who experienced attack was slightly higher, ranging from 22 to 32% depending on the year. Considering HAE-related hospitalizations, the proportion of patients who had at least one admission ranged from 23 to 56% depending on the year after danazol initiation. In the present study, the proportion of patients with a record of ICU admission ranged from 2 to 8% when considered HAE-related ICU admissions. Although all patients had at least one ambulatory visit in the first year after danazol, only 15% of the ambulatory care were HAE-related in the first year, with lower proportions across the following years. Several patients with low-to-moderate symptoms (acute swelling) that search for ambulatory assistance might not be detected in the database due to the limitation on registry signs and symptoms and their ICD-10 code related to these types of clinical manifestations.

The 2015–2016 Nationwide Emergency Department Sample from the United States involving HAE patients shows that 8.2% of HAE patients were hospitalized (24). A real-world study with HAE patients in the United States between 2006 and 2014 under prophylactic or on-demand treatment showed that 15% of patients experienced one or more hospitalizations, and 52% had one or more emergency visits during the study period (30) and other evidence showed that HAE accounts for 15,000–30,000 emergency room visits each year in the United States (11). These numbers account for diagnosed patients and could be even higher. Previous studies showed that HAE abdominal pain presents similarly to and is often mistaken for appendicitis, bowel obstruction, or other gastrointestinal diseases resulting in unnecessary image procedures and even surgeries, such as appendicectomy, cholecystectomy, and hysterectomy (24). A case report study in Japan mentioned that a woman had a medical history of abdominal attacks at the hospital 2 or 3 times a year with a misdiagnosis of enteritis and endometriosis. The same women also presented 3 episodes of life-threatening laryngeal edema, 2 of which were unsuccessfully treated with steroids and antihistamines (31). In one study developed in Brazil, 50% of patients had at least one episode of laryngeal edema, and almost 27% were mistakenly submitted to laparotomy (11). Considering these aspects, we may hypothesize that if the proper diagnosis was made, the hospitalization and HCRU would be higher in our study.

### 4.1 Limitations

It is important to note that for the present study, to identify attacks within the HAE cohort, a list of ICD-10 codes (Supplementary Table S2) and procedures (Supplementary Table S3) that could potentially be related to HAE attacks (i.e., ICD-10 codes and procedures most commonly presented as symptoms/manifestations of HAE attacks) was created based on the literature review and clinical expert. Any ICD-10 records and/or procedures listed in these tables were considered as an indicator/proxy for attacks. Finally, for analysis purposes, the list of ICD-10 codes and procedures pre-defined as an attack was segregated into severe and non-severe attacks. An even more restricted list of procedures and ICD-10 codes were used to identify HAE patients with severe disease.

The higher number of new HAE cases found in 2011 and the lower number in 2021 have an important influence on the study design: 2011, as the first year of the study, may also include patients who have had the first claim of danazol prior to this date, and 2021, as the last year of the study, included only patients who have had the first claim of danazol prior to June 2021 as the eligibility criteria describe the necessity to have more than 6 months of information available in the database after the danazol initiation. Moreover, the number of patients during the last 2 years (2020 and 2021) may have been affected by the COVID-19 pandemic due the decreased number of patients searching for health assistance in this period. The only HAE patient’s registry available in Brazil is from the Brazilian Association of Patients with HAE (ABRANGHE) which is based on data from self-registry. They have identified a total of 1,679 cases of HAE across the country, but there is no distinguishing if patients are using or not danazol and/or searching for public or private assistance which may justify the differences found.

The major limitation of retrospective secondary database studies is the data incompleteness which depends on the quality of filling of non-mandatory data. Hereditary angioedema disease does not have a specific ICD-10 code, and the DATASUS uses it to identify the respective disease. In addition, due to the administrative character of the database used, little clinical information was available, so the only specific predictive variables for identifying HAE cases and their attacks were ICD-10 codes and procedures. Aiming to reduce the probability of including patients with other diseases than HAE, we have defined very specific mandatory procedures for classifying HAE cases and a very broad list of potential confounding diseases as exclusion criteria. Together, this might have an impact in the number of patients identified as HAE and the number of attacks and healthcare resources found in the present study. Second, we believe that the possibility to be diagnosed or even raising the possibility to be HAE in patients with clinical manifestations such as intestinal, laryngeal edema, or other common clinical manifestations of HAE is very likely to be restricted to large centers, as many physicians may not be familiar with HAE and its attacks, which can result in a lower number of patients diagnosed and treated. Thus, our data and literature showed that HAE patients pose a considerable burden on patients, their families, and healthcare systems in terms of both direct and indirect costs at the time of attacks and in between them. Finally, DATASUS captures information from the public health system in Brazil, which might represent 100% of the Brazilian population, although other supplementary health insurance might be used for the population and it is not represented in the present study (32).

## 5 Conclusion

This database study is the first of its kind and the developed strategy applied to find patients with HAE who use danazol for long-term prophylaxis allowed us to describe the characteristics of patients in the SUS and also identify HCRU outcomes of interest such as hospitalizations, inpatient, and outpatient settings. This study also allowed us to assess the frequency of attacks and severe attacks among those who needed to use resources from public institutions conditioned to inpatient and outpatient settings.

Our findings also highlighted that even with limitations due to the study design, we could capture the attack’s prevalence of more than 30 and 4% of life-threatening severe attack that included asphyxia and edema laryngeal in addition to surgeries and other invasive procedures performed due to attacks.

The important number of attacks, hospitalizations, and general resource uses highlights the necessity to increase awareness of new strategies and accurate approaches to treat HAE patients. Therefore, our findings are important indicators that our health system and guidelines need to be revised and improved to properly diagnose, treat, and assist patients with HAE.

## Data availability statement

The original contributions presented in the study are included in the article/Supplementary material, further inquiries can be directed to the corresponding author.

## Ethics statement

Ethical approval and written informed consent was not required for study that only use publicly and anonymized secondary database in accordance with the local legislation and institutional requirements.

## Author contributions

AR: Conceptualization, Data curation, Formal analysis, Writing – original draft, Investigation, Methodology, Project administration, Resources, Software, Visualization. SS: Conceptualization, Formal analysis, Writing – original draft, Data curation, Investigation, Methodology, Resources, Software, Visualization. RP: Conceptualization, Writing – review & editing, Funding acquisition, Supervision. JS: Conceptualization, Writing – review & editing, Funding acquisition, Supervision. FC: Conceptualization, Writing – review & editing, Funding acquisition, Project administration, Supervision. TR: Data curation, Formal analysis, Conceptualization, Writing – review & editing, Investigation, Methodology, Resources. SV: Formal analysis, Conceptualization, Writing – review & editing, Data curation, Methodology, Project administration, Resources, Visualization.

## Glossary

ABRANGHE: Brazilian Association of Patients with HAE
ACEi: Angiotensin-converting-enzyme inhibitors
ARBs: Angiotensin receptor blockers
C1-INH: C1 inhibitor
CI: Confidence Interval
DATASUS: Informatics Department of Unified Health System
HAE: Hereditary angioedema
HCRU: Healthcare Resource Utilization
ICD-10: International Classification of Diseases 10^th^ revision
ICU: Intensive Care Unit
IQR: Interquartile Range
NSAID: Non-steroidal anti-inflammatory drugs
PCDT: Clinical Protocols and Therapeutic Guidelines
pdC1-INH: Plasma-derived human C1-INH
PPPY: Per patient per year
RWD: Real-World Data
RWE: Real-World Evidence
SD: Standard Deviation
SIA: Outpatient Information System
SIGTAP: Procedures, Medications and Orthoses, Prostheses and Special Materials Table Management System
SIH: Inpatient Information System
SUS: Brazilian Public Healthcare System
