# Supplementary material for: A real-world study of hereditary angioedema patients due to C1 inhibitor deficiency treated with danazol in the Brazilian Public Health System
Source: Front Med (Lausanne). 2024 Sep 6;11:1343547. doi: 10.3389/fmed.2024.1343547 (PMC11414478; doi:10.3389/fmed.2024.1343547)
Supplement: Supplementary file 1 [file Data_Sheet_1.docx]

Supplementary Material

**A Real-World Study of Hereditary Angioedema Patients due to C1 inhibitor deficiency Treated with Danazol in the Brazilian Public Health System**

Alessandra Mileni Versuti Ritter^2^, Suelen Silva^2^, Robson de Paula^1^, Juliana Senra^1^, Fabio Carvalho^1^, Tatiane Ribeiro^1^, Solange Oliveira Rodrigues Valle^3^

^1^Takeda Distribuidora Ltd., São Paulo Brazil

^2^IQVIA, São Paulo, Brazil

^3^Hospital Universitário Clementino Fraga Filho da Universidade Federal do Rio de Janeiro, Rio de Janeiro, RJ, Brazil

*** Correspondence:**Solange Oliveira Rodrigues Valle
solangervalle@gmail.com

**Supplementary Tables**

**Table I.** List of ICD-10 potentially resulting in misclassification.

| **ICD-10 CODE** | **Description** |
| --- | --- |
|  |  |
| **Q82.0** | Hereditary lymphoedema |
| **I89.0** | Lymphoedema, not elsewhere classified |
| **G51.2** | Melkersson syndrome |
| **D68.1** | Hereditary factor XI deficiency |
| **Q18.6** | Macrocheilia |
| **D69.3** | Idiopathic thrombocytopenic purpura |
| **L930** | Discoid lupus erythematosus |
| **L931** | Subacute cutaneous lupus erythematosus |
| **M321** | Systemic lupus erythematosus with organ or system involvement |
| **M328** | Other forms of systemic lupus erythematosus |
| **N800** | Endometriosis of uterus |
| **N801** | Endometriosis of ovary |
| **N802** | Endometriosis of fallopian tube |
| **N803** | Endometriosis of pelvic peritoneum |
| **N804** | Endometriosis of rectovaginal septum and vagina |
| **N805** | Endometriosis of intestine |
| **N808** | Other endometriosis |
| **L50.0** | Allergic urticaria |
| **L50.1** | Idiopathic urticaria |
| **L50.2** | Urticaria due to cold and heat |
| **L50.3** | Dermatographic urticaria |
| **L50.4** | Vibratory urticaria |
| **L50.5** | Cholinergic urticaria |
| **L50.6** | Contact urticaria |
| **L50.8** | Other urticaria |
| **L50.9** | Urticaria, unspecified |
| **D47** | Other neoplasms of uncertain or unknown behavior of lymphoid, hematopoietic and related tissue |
| **D47.2** | Monoclonal gammopathy of undetermined significance (MGUS) |
| **C81** | Hodgkin lymphoma |
| **C81.0** | Nodular lymphocyte predominant Hodgkin lymphoma |
| **C81.1** | Nodular sclerosis (classical) Hodgkin lymphoma |
| **C81.2** | Mixed cellularity (classical) Hodgkin lymphoma |
| **C81.3** | Lymphocyte depleted (classical) Hodgkin lymphoma |
| **C81.4** | Lymphocyte-rich (classical) Hodgkin lymphoma |
| **C81.7** | Other (classical) Hodgkin lymphoma |
| **C81.9** | Hodgkin lymphoma, unspecified |
| **C82** | Follicular lymphoma |
| **C82.0** | Follicular lymphoma grade I |
| **C82.1** | Follicular lymphoma grade II |
| **C82.2** | Follicular lymphoma grade III, unspecified |
| **C82.3** | Follicular lymphoma grade IIIa |
| **C82.4** | Follicular lymphoma grade IIIb |
| **C82.5** | Diffuse follicle centre lymphoma |
| **C82.6** | Cutaneous follicle centre lymphoma |
| **C82.7** | Other types of follicular lymphoma |
| **C82.9** | Follicular lymphoma, unspecified |
| **C83** | Non-follicular lymphoma |
| **C83.0** | Small cell B-cell lymphoma |
| **C83.1** | Mantle cell lymphoma |
| **C83.3** | Diffuse large B-cell lymphoma |
| **C83.5** | Lymphoblastic diffuse lymphoma |
| **C83.7** | Burkitt lymphoma |
| **C83.8** | Other non-follicular lymphoma |
| **C83.9** | Non-follicular (diffuse) lymphoma, unspecified |
| **C84** | Mature T/NK-cell lymphomas |
| **C84.4** | Peripheral T-cell lymphoma, not elsewhere classified |
| **C84.5** | Other mature T/NK-cell lymphomas |
| **C84.8** | Cutaneous T-cell lymphoma, unspecified |
| **C84.9** | Mature T/NK-cell lymphoma, unspecified |
| **C85** | Other and unspecified types of non-Hodgkin lymphoma |
| **C85.1** | B-cell lymphoma, unspecified |
| **C85.2** | Mediastinal (thymic) large B-cell lymphoma |
| **C85.7** | Other specified types of non-Hodgkin lymphoma |
| **C85.9** | Non-Hodgkin lymphoma, unspecified |
| **C86** | Other specified types of T/NK-cell lymphoma |
| **C86.1** | Hepatosplenic T-cell lymphoma |
| **C86.2** | Enteropathy-type (intestinal) T-cell lymphoma |
| **C86.3** | Subcutaneous panniculitis-like T-cell lymphoma |
| **C86.6** | Primary cutaneous CD30-positive T-cell proliferations |
| **C88** | Malignant immunoproliferative diseases |
| **C88.0** | Waldenström macroglobulinemia |
| **C88.4** | Extranodal marginal zone B-cell lymphoma of mucosa-associated lymphoid tissue [MALT-lymphoma] |
| **C88.7** | Other malignant immunoproliferative diseases |
| **C88.9** | Malignant immunoproliferative disease, unspecified |
| **C90** | Multiple myeloma and malignant plasma cell neoplasms |
| **C90.0** | Multiple myeloma |
| **C91** | Lymphoid leukaemia |
| **C91.0** | Acute lymphoblastic leukaemia |
| **C91.1** | Chronic lymphocytic leukaemia of B-cell type |
| **C91.8** | Mature B-cell leukaemia Burkitt-type |
| **C91.4** | Hairy-cell leukaemia |
| **D72.1** | Eosinophilia |
| **D76.1** | Hemophagocytic lymphohistiocytosis |
| **D82.0** | Wiskott-Aldrich syndrome |
| **D82.3** | Immunodeficiency following hereditary defective response to Epstein-Barr virus |
| **J84.2** | Lymphoid interstitial pneumonia |
| **L41.0** | Pityriasis lichenoides et varioliformis acuta |
| **L41.1** | Pityriasis lichenoides chronica |

**Table II.** International Classification of Diseases (ICD-10) code list for identifying HAE attacks cases in DATASUS.

| **ICD-10 CODE** | **Description** |
| --- | --- |
| **K35.8** | Acute appendicitis, other and unspecified |
| **K36** | Other appendicitis |
| **K37** | Unspecified appendicitis |
| **K58** | Irritable bowel syndrome |
| **R10.0** | Acute abdomen (abdominal and pelvic pain) |
| **R09.0** | Asphyxia |
| **J38.4** | Oedema of larynx |
| **K85** | Acute pancreatitis |
| **T78.2** | Anaphylactic shock, unspecified |
| **T78.3** | Angioneurotic edema |
| **R49.0** | Dysphonia |
| **R60** | Oedema, not elsewhere classified |
| **T78.4** | Allergy, unspecified |
| **R52.1** | Chronic intractable pain |
| **R52.2** | Other chronic pain |
| **R52.9** | Pain, unspecified |
| **R10** | Abdominal pain |
| **L53.2** | Erythema marginatum |
| **R60.1** | Stridor |
| **R06.8** | Breathlessness |
| **R60** | Oedema |
| **R52.0** | Acute pain |
| **R10.4** | Other and unspecified abdominal pain |
| **R60.9** | Oedema, unspecified |
| **J039** | Acute tonsillitis, unspecified |
| **L98** | Other infections of skin classified in other parties |
| **R22** | Localized swelling, mass and lump of skin and subcutaneous tissue |
| **R19.0** | Intra-abdominal and pelvic swelling, mass and lump |
| **T88.4** | Failed or difficult intubation |
| **J96.0** | Acute respiratory failure |
| **J96.9** | Acute respiratory failure not specific |
| **J98.8** | Other specific respiratory disorders |
| **J98.9** | Non-specific respiratory disorders |

**Table III.** List of SUS standard procedures potentially related to HAE attacks selected for the study.

| **SIGTAP Code** | **Procedure** |
| --- | --- |
| **0209010045** | Laparoscopy |
| **0306020106** | Plasma transfusion |
| **0301100071** | Tracheostomy care |
| **0404010377** | Tracheostomy |
| **0412010127** | Tracheostomy with placement of a tracheal orthosis |
| **0412020076** | Mediastinal tracheostomy |
| **0702050024** | Cannula for tracheostomy |
| **0303060050** | Anaphylactic shock treatment |
| **0209010061** | Videolaparoscopy |
| **0303070099** | Treatment of enteritis and non-infectious colitis |
| **0303070102** | Treatment of other diseases of digestive tract |
| **0303070110** | Treatment of other bowel diseases |
| **0303140127** | Treatment of other upper respiratory diseases |
| **0303140135** | Treatment of other respiratory tract diseases |
| **0302040013** | Physiotherapeutic care in patients with systemic respiratory disorder |
| **0302040021** | Physiotherapeutic care in patients with non-systemic respiratory disorder |

**Table IV.** List of SUS standard procedures potentially related to HAE severe attacks.

| **ICD-10 CODE** | **Description** |
| --- | --- |
| **R09.0** | Asphyxia |
| **J38.4** | Oedema of larynx |
| **K85** | Acute pancreatitis |
| **T78.2** | Anaphylactic shock, unspecified |
| **R60.1** | Stridor |
| **R06.8** | Breathlessness |
| **Procedure code** | **Description** |
| **0209010045** | Laparoscopy |
| **0301100071** | Tracheostomy care |
| **0404010377** | Tracheostomy |
| **0412010127** | Tracheostomy with placement of a tracheal orthosis |
| **0412020076** | Mediastinal tracheostomy |
| **0702050024** | Cannula for tracheostomy |
| **0303060050** | Anaphylactic shock treatment |
| **0209010061** | Videolaparoscopy |
| **0302040013** | Physiotherapeutic care in patients with systemic respiratory disorder |
| **0302040021** | Physiotherapeutic care in patients with non-systemic respiratory disorder |

**Table V.** Proportion of patients with severe attacks (report of ICD-10 and/or procedures claim defined as proxy of severe attack*) by calendar year (n=799).

|  | **2011** | **2012** | **2013** | **2014** | **2015** | **2016** | | **2017** | **2018** | **2019** | **2020** | **2021** |
| --- | --- | --- | --- | --- | --- | --- | --- | --- | --- | --- | --- | --- |
| **Total population with attendance by year** | n= 379 | n= 440 | n= 483 | n=512 | n=506 | n=407 | | n=361 | n=413 | n=381 | n=290 | n=322 |
| **Patients with severe attack in the respective year, among the total population attended per year – n (%)** | 4 (1.1) | 5 (1.1%) | 2 (0.4%) | 3 (0.6%) | 4 (0.8%) | 2 (0.5%) | 3 (0.8%) | | 3 (0.7%) | 4 (1.1%) | 2 (0.7%) | 3 (0.9%) |
| **Severe attack population with attendance by year** | **14** | **18** | **19** | **21** | **22** | **18** | **18** | | **18** | **21** | **16** | **15** |
| **Patients with severe attack in the respective year - n (%)** | 4 (28.6%) | 5 (27.8%) | 2 (10.5%) | 3 (14.3%) | 4 (18.2%) | 2 (11.2%) | 3 (16.7%) | | 3 (16.7%) | 4 (19.0%) | 2 (12.5%) | 3 (20.0%) |
| Patients with 1 attack | 3 (21.4%) | 4 (22.2%) | 2 (10.5%) | 2 (9.5%) | 2 (9.1%) | 1 (5.6%) | 2 (11.1%) | | 2 (11.1%) | 3 (14.3%) | 2 (12.5%) | 2 (13.3%) |
| Patients with 2 attacks | 0 (0.0%) | 1 (5.6%) | 0 (0.0%) | 0 (0.0%) | 1 (4.6%) | 0 (0.0%) | 1 (5.6%) | | 0 (0.0%) | 1 (4.8%) | 0 (0.0%) | 0 (0.0%) |
| Patients with 3 attacks | 1 (7.1%) | 0 (0.0%) | 0 (0.0%) | 1 (4.8%) | 0 (0.0%) | 0 (0.0%) | 0 (0.0%) | | 0 (0.0%) | 0 (0.0%) | 0 (0.0%) | 0 (0.0%) |
| Patients with 4 attacks | 0 (0.0%) | 0 (0.0%) | 0 (0.0%) | 0 (0.0%) | 0 (0.0%) | 0 (0.0%) | 0 (0.0%) | | 0 (0.0%) | 0 (0.0%) | 0 (0.0%) | 0 (0.0%) |
| Patients with 5 attacks | 0 (0.0%) | 0 (0.0%) | 0 (0.0%) | 0 (0.0%) | 0 (0.0%) | 0 (0.0%) | 0 (0.0%) | | 0 (0.0%) | 0 (0.0%) | 0 (0.0%) | 0 (0.0%) |
| Patients with 6 attacks | 0 (0.0%) | 0 (0.0%) | 0 (0.0%) | 0 (0.0%) | 0 (0.0%) | 0 (0.0%) | 0 (0.0%) | | 0 (0.0%) | 0 (0.0%) | 0 (0.0%) | 1 (6.7%) |
| Patients with 7 attacks | 0 (0.0%) | 0 (0.0%) | 0 (0.0%) | 0 (0.0%) | 0 (0.0%) | 0 (0.0%) | 0 (0.0%) | | 0 (0.0%) | 0 (0.0%) | 0 (0.0%) | 0 (0.0%) |
| Patients with 8 or more attacks | 0 (0.0%) | 0 (0.0%) | 0 (0.0%) | 0 (0.0%) | 1 (4.6%) | 1 (5.6%) | 0 (0.0%) | | 1 (5.6%) | 0 (0.0%) | 0 (0.0%) | 0 (0.0%) |
| **General hospitalization – n of patients (%)** | 3 (21.4%) | 5 (27.8%) | 4 (21.1%) | 7 (33.3%) | 11 (50.0%) | 8 (44.4%) | 8 (44.4%) | | 7 (38.9%) | 10 (47.6%) | 10 (62.5%) | 5 (33.3%) |
| **General ICU hospitalization – n of patients (%)** | 1 (7.1%) | 2 (11.1%) | 3 (15.8%) | 4 (19.1%) | 4 (18.2%) | 4 (22.2%) | 3 (16.7%) | | 1 (5.6%) | 4 (19.1%) | 4 (25.0%) | 2 (13.3%) |
| **ICD-10 HAE-related ICU – n of patients (%)** | 0 (0.0%) | 0 (0.0%) | 0 (0.0%) | 0 (0.0%) | 0 (0.0%) | 0 (0.0%) | 1 (5.6%) | | 0 (0.0%) | 0 (0.0%) | 1 (6.3%) | 0 (0.0%) |
| Anaphylactic shock treatment | 1 (25.0%) | 2 (40.0%) | 1 (50.0%) | 1 (33.33%) | 0 (0.0%) | 0 (0.0%) | 2 (66.7%) | | 1 (33.3%) | 3 (75.0%) | 1 (50.0%) | 1 (33.3%) |
| Tracheostomy | 2 (50.0%) | 2 (40.0%) | 1 (50.0%) | 1 (33.3%) | 0 (0.0%) | 0 (0.0%) | 0 (0.0%) | | 0 (0.0%) | 0 (0.0%) | 1 (50.0%) | 0 (0.0%) |
| Physiotherapeutic care in patients with non-systemic respiratory disorder | 1 (25.0%) | 1 (20.0%) | 0 (0.0%) | 1 (33.3%) | 2 (50.0%) | 1 (50.0%) | 1 (33.3%) | | 0 (0.0%) | 0 (0.0%) | 0 (0.0%) | 2 (66.7%) |
| Physiotherapeutic care in patients with systemic respiratory disorder | 0 (0.0%) | 0 (0.0%) | 0 (0.0%) | 0 (0.0%) | 0 (0.0%) | 0 (0.0%) | 0 (0.0%) | | 1 (33.3%) | 0 (0.0%) | 0 (0.0%) | 0 (0.0%) |
| **ICD-10 HAE-related hospitalization – n of patients (%)** | 1 (7.1%) | 0 (0.0%) | 0 (0.0%) | 0 (0.0%) | 2 (9.1%) | 1 (5.6%) | 2 (11.1%) | | 1 (5.6%) | 1 (4.8%) | 1 (6.3%) | 0 (0.0%) |
| Anaphylactic shock, unspecified | 0 (0.0%) | 1 (20.0%) | 1 (50.0%) | 0 (0.0%) | 0 (0.0%) | 0 (0.0%) | 2 (66.7%) | | 1 (50.0%) | 1 (33.3%) | 1 (50.0%) | 0 (0.0%) |
| Breathlessness | 0 (0.0%) | 0 (0.0%) | 0 (0.0%) | 0 (0.0%) | 0 (0.0%) | 0 (0.0%) | 0 (0.0%) | | 0 (0.0%) | 0 (0.0%) | 0 (0.0%) | 0 (0.0%) |
| Oedema of larynx | 1 (25.0%) | 0 (0.0%) | 0 (0.0%) | 0 (0.0%) | 0 (0.0%) | 0 (0.0%) | 0 (0.0%) | | 0 (0.0%) | 0 (0.0%) | 0 (0.0%) | 0 (0.0%) |
